# Vascular dementia: Current concepts and nomenclature harmonization

**DOI:** 10.1590/S1980-57642012DN06030002

**Published:** 2012

**Authors:** Lea Tenenholz Grinberg

**Affiliations:** Department of Neurology, University of California San Francisco - 675 Nelson Rising Lane, San Francisco – CA – 94158 – USA. Departamento de Patologia da FMUSP – Av. Dr. Arnaldo,455 / sala 1353 – 01246903 São Paulo SP, Brazil.

**Keywords:** pathology, Alzheimer's disease, cerebrovascular diseases, vascular dementia

## Abstract

Several types of cerebrovascular lesions are associated with cognitive decline, but the role of each type in dementia manifestation has yet to be determined. One of the greatest barriers of conducting clinicopathological studies in vascular dementia concerns the overlapping of nomenclature for these lesions. The aim of the present review was to discuss current nomenclature for cerebrovascular lesions and suggest modifications to allow better diagnostic reproducibility in this field

## Abstract (pt)

Diversas lesões cerebrovasculares estão associadas à perda cognitiva. Entretanto ainda não se conhece qual a contribuição exata dessas lesões para quadros os demenciais. Um dos maiores empecilhos para estudos de correlação clinicopatológica em demências vasculares é a heteregeonidade de termos usados para definir essas lesões. A presente revisão discute a nomenclatura neuropatológica atual para lesões cerebrovasculares e sugere alternativas para melhorar a acurácia e reprodutividade do diagnóstico das mesmas.

## INTRODUCTION

Cerebral arteriosclerosis was considered the main cause of senile dementia in the beginning of the twentieth century.^1^ However, the demonstration of Alzheimer's disease (AD) type as very frequent in control elderly and demented subjects led to such an extreme shift in this view that for many decades dementia of vascular origin was virtually dismissed in the differential diagnosis of dementia. More recently, vascular dementia again became a focus of attention following clinicopathological studies showing that even a small degree of vascular brain change may cause cognitive decline if occurring in a strategic area.^2-3^

One of the most important barriers preventing further advances in the understanding of vascular dementia is a lack of a reliable way of determining which element of the cognitive decline is due to vascular changes. Unlike other common causes of dementia, vascular dementia is not easily identifiable by a specific neuropathological hallmark, such as neuritic plaques in AD or Lewy bodies in Parkinson's disease. In addition, vascular changes are also found frequently in brains of cognitively normal elderly,^4^ making it difficult to establish a causal relationship between brain lesions and cognitive decline. Moreover, vascular changes are likely to have a synergistic rather than an additive effect to primary neurodegenerative processes.^5-8^ Therefore, a pathological diagnosis of vascular dementia (VaD) is often reached by excluding other causes.

A drastic change in the way cerebrovascular lesions are defined is critical for the creation of a new set of pathological diagnostic criteria.^2^ Vascular dementia is not a single entity, but an umbrella term to describe cognitive decline due to a series of different vessel disorders, frequently seen in combination with other non-vascular changes. These vessel disorders can induce various types of cerebral tissue lesions such as hemorrhage, infarction, hippocampal sclerosis, and white matter lesions.^9^

Currently, vascular lesions are classified based on their morphological characteristics rather than by their pathogenesis. The aims of this review article were to review characteristics of cerebrovascular lesions associated with cognitive decline, discuss overlapping nomenclature and propose strategies to harmonize the nomenclature adopted for vascular brain disorders.

## CURRENT CLASSIFICATION AND NOMENCLATURE OF VASCULAR BRAIN DISORDERS

Current classifications distinguish vessel disorders from parenchymal lesions. There is a great deal of overlapping among the different categories.^10-12,14^

## VESSEL DISORDERS

Vessel disorders are divided into those involving large or small vessels.

**Large vessel disorders.** Atherosclerosis refers to age-related degenerative vessel disorder of medium- to large-sized arteries, in which the circle of Willis is the most vulnerable site. Atherosclerosis progression follows a predictive sequence starting with intima proliferation and accumulation of blood-derived lipids and proteins, especially cholesterol within vessel walls, resulting in atherosclerotic plaques and further degeneration and fibrosis of vessel walls.^15,16^ Atherosclerotic plaques often break up leading to thrombosis or emboli.^9,17^

**Figure 1:**
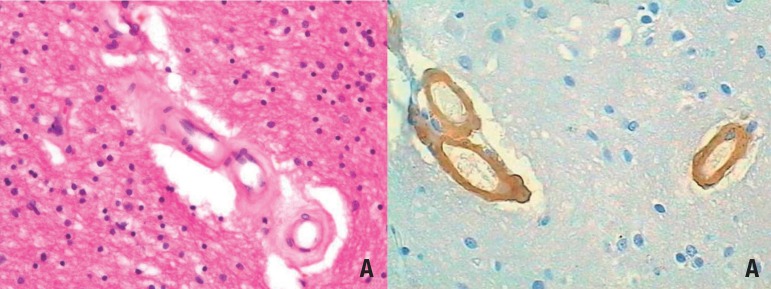
Example of small vessel disease. [A] Subcortical white matter exhibiting hyaline arteriolosclerosis. Note how the arterial wall is thickened by hyaline material. HE. 200x. [B] Cortex exhibiting cerebral amyloid angiopathy. Amyloid-β peptide infiltrates the vessel wall. 200x. Immunohistochemistry against amyloid-β.

**Small vessel disorders (Figure 1).** Small vessel disease (SVD) encompasses distinct changes such as small vessel arteriosclerosis, arteriolosclerosis, arteriohyalinosis, and lipohyalinosis.^9,18,19^ Arteriosclerosis resembles large vessel atherosclerosis, with the exception of calcification.^12,20^ Arteriolosclerosis occurs by concentric hyaline thickening of vessel walls with stenosis of arterioles.^20^ Lipohyalinosis is characterized by asymmetric areas of fibrosis, hyalinosis associated with foam cells, and accumulation of blood-derived lipids and proteins.^9,21,22^ White matter arteries often show loss of smooth muscle cells, fibrosis, and thickening of the basement membrane, as well as enlarged perivascular spaces with leakage of plasma proteins.^23^

Preferential sites of involvement for SVD are basal ganglia, followed by peripheral white matter and leptomeningeal arteries, thalamus and cerebellar white matter. Cortical vessels are usually spared.^24^ SVD is an important cause of white matter destruction^25,26^ and Wallerian degeneration.^27^ More details about SVD progression can be found elsewhere.^24^

Sporadic cerebral amyloid angiopathy (CAA) is characterized by amyloid protein, mainly amyloid-β 1-40, deposits in cerebral and leptomeningeal artery, vein and capillary walls. Typically, these deposits are located near the basement membrane or in the smooth muscle cell layer.^28,29^ CAA rarely leads to lethal hemorrhage,^28^ and more often to microbleeds,^30^ capillary occlusion, blood flow disturbances^31^ and microinfarcts.^32^ CAA is frequently found together with AD-type changes, but this finding is not universal.

## VASCULAR-ASSOCIATED PARENCHYMAL LESIONS COMMONLY SEEN IN COGNITIVE DECLINE

Parenchymal disorders are mainly divided into ischemic or hemorrhagic.

**Ischemic.** Brain infarcts are subclassified by size into large (greater than 1.5 cm^3^ or 1 cm in diameter), lacunar (0.5-1.^5^ cm^3^ in volume or 0.5-1.0 cm in diameter) and microinfarcts (not seen macroscopically, usually less than 0.5 cm in diameter).^9,12^

**Figure 2:**
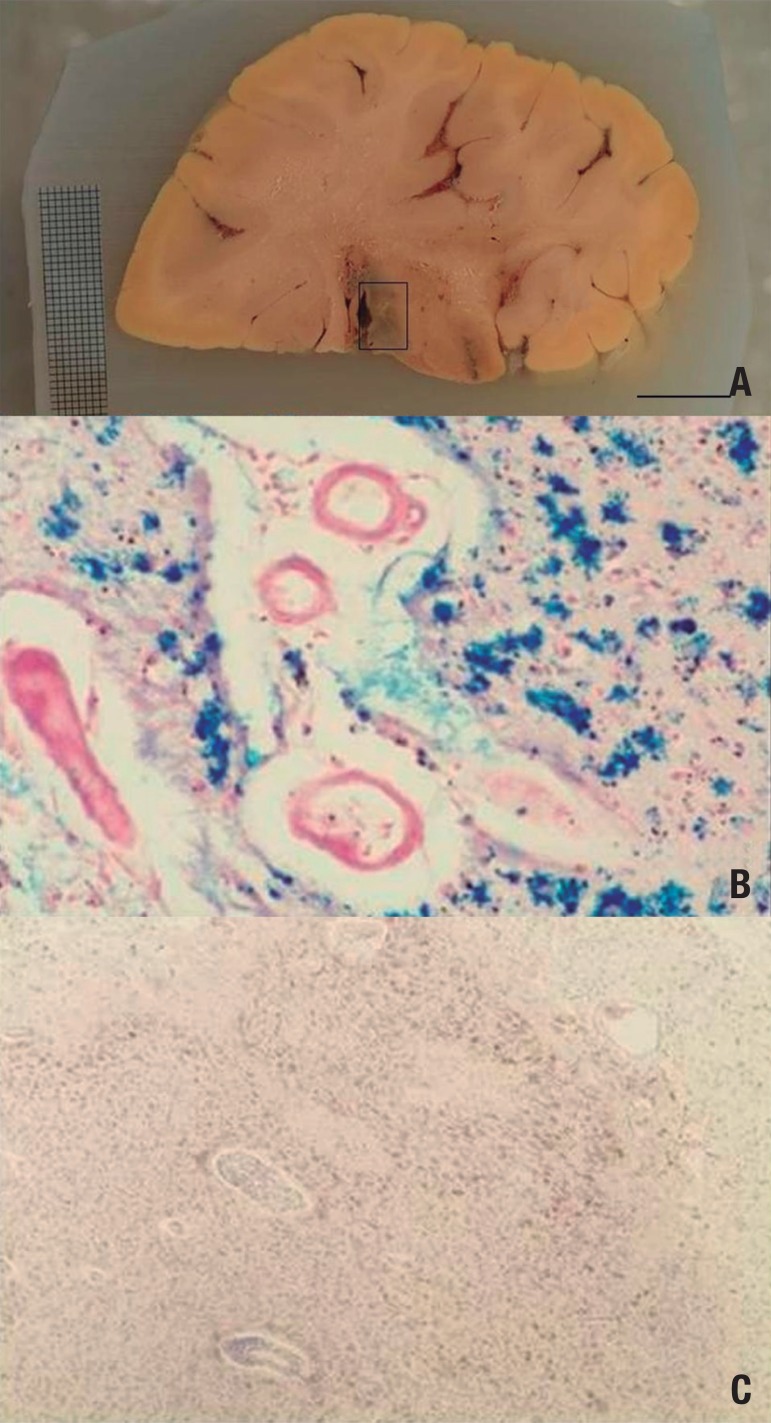
Hemorrhagic infarcts. [A] Coronal section across the thalamus. The boxed area encompasses an hemorrhagic infarct. Note how the tissue color is darker at this site due to hemosiderin. [B] Histological slide of the infarcted area stained for iron. The blue staining represents iron deposits, an indirect marker of bleeding. [C] Histological slide of the infarcted area stained with HE. The whole area is saturated by hemosiderin (in brown).

Ischemic infarcts (Figure 2) represent the majority of large infarcts, and usually follow artery occlusion. Infarcts located between the territories of two major arteries are called watershed or borderzone infarcts. Clinical manifestation depends on location and ranges from motor impairment to language and cognitive difficulties.^33^ A recent meta-analysis showed that 10% of stroke patients had dementia before their first episode, and more than a third developed dementia after recurrent strokes.^34^

Lacunar infarcts are visible radiologically and upon gross examination. They are largely confined to cerebral white matter and subcortical structures because the lack of anastomoses makes these regions more vulnerable. It is believed, but not proven, that lacunar infarcts are either caused by SVD-related vessel occlusion or by embolic events. Certainly, lacunar infarcts are associated with hypertension^9^ and may evolve with cognitive decline.^35,36^ The special terms *etat lacunaire* or *status lacunaris* (when seen in gray matter) and *etat crible* or *status cribrosus* (when seen in white matter) indicate a large number of lacunes in the same region, and have no pathogenic meaning.^37^

Microinfarcts are often associated with SVD and CAA, but can also be caused by thromboembolism.^38,39^ They are invisible on gross or imaging examinations and most commonly found in the watershed areas of cortex. Besides their apparent irrelevance, microinfarcts contribute to cognitive decline.^40^

Cortical laminar necrosis or pseudolaminar necrosis is characterized by neuronal loss and gliosis in the neocortex as a consequence of global hypotension or hypoxaemia. Therefore, they are most evident at arterial borderzones.^41^

Hippocampal sclerosis (HS) describes abundant neuronal loss without pseudocystic cavitation in the CA1 sector of the hippocampus and subiculum. Although these cells are very sensitive to ischemia, making it logical to associate cognitive decline to vascular problems, they predominate in epilepsy cases, while HS-associated frontotemporal lobar degeneration is the form most frequently seen in dementia.^42^

White matter lesions are found in up to 65% of subjects over 65 years of age, and their frequency increases in patients with cerebrovascular disease or cardiovascular risk. WMLs usually comprise, to varying degrees, demyelination, axonal loss, mild reactive astrocytosis, edema, macrophage reaction, and microangiopathy of the penetrating arteries. As a rule, the subcortical U-fibers are spared.^26,27,43^ Binswanger was the first to suggest that such changes evolve with cognitive impairment.^44^

**Hemorrhagic.** Hemorrhagic infarcts occur after reperfusion of an ischemic infarct or when remaining or collateral blood flow is insufficient to keep the infarcted area viable and spills into the damaged area.^9^ Ischemic damage of the vessel walls in the infarct area and impaired coagulation mechanisms (e.g. lysis therapy) may facilitate blood leakage under the above-mentioned conditions leading to hemorrhagic infarcts.

Cerebral hemorrhage is a massive blood influx into an intact brain parenchyma after vessel rupture. Note that infarcts and hemorrhaging are two different processes. Large hemorrhages displace brain tissue and are often fatal due to brain edema, increased intracranial pressure and herniations. Hypertension in arteries involved by SVD is the most frequent cause of cerebral hemorrhage, followed by CAA.^45-48^ Aneurysms and vascular malformations rarely cause hemorrhage in the elderly. Cognition related brain areas are usually affected in this process.

Microbleed is the term used to describe either blood leakage into perivascular or Virchow-Robin spaces, or small intracerebral hemorrhages measuring less than 10 mm in diameter.^49^ Microbleeds are age-related and believed to be surrogates of microvascular disease, but their exact pathogenesis and cognitive impact has yet to be clarified^50,51^ Radiologically, microbleeds can be easily detected by magnetic resonance imaging as areas of signal loss.^30^ As such, radiologically-detected microbleeds in the cortex are indicative of CAA whereas those seen in white matter point to SVD.^52^ Caution should be taken in interpreting imaging results, since it has been demonstrated that striatal microbleeds are overestimated even on 7.0 T MRI.^51^

## STRATEGIES FOR ALLOWING HARMONIZATION OF TERMINOLOGY FOR VESSEL DISORDERS AND THEIR ASSOCIATED TISSUE LESIONS

Current terminology for cerebrovascular lesions is based on descriptive characteristics, and has a great deal of overlap. For instance, atherosclerotic lesions in small arteries can be defined either as atherosclerosis or as small vessel disease. Lacunar infarcts are by definition smaller than 1.0 cm, but giant lacunae are described in the literature. Moreover, lacunar infarcts, lacunar hemorrhages, and enlarged perivascular spaces with lacunar appearance are all termed lacunes by some authors, despite the fact they may be caused by different processes.^17^

**Figure 3:**
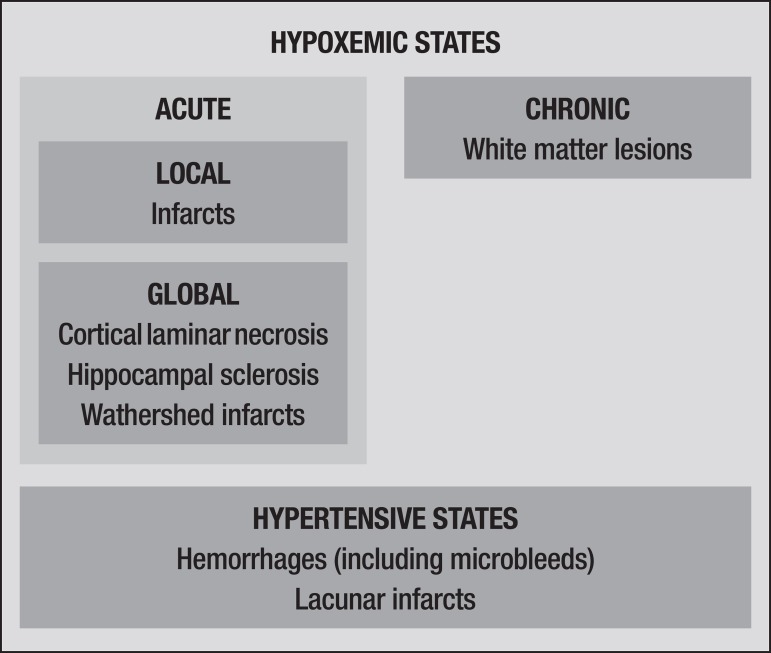
Suggested pathogenesis-based classification for cerebrovascular lesions.

A classification based on pathogenesis is likely to ameliorate overlapping problems and enable a more readily reproducible set of diagnosis criteria. For instance, hypoxemic lesions should be classified as global or local. This scheme would place ischemic infarcts and watershed infarcts in different categories (Figure 3).

An important prerequisite is required before attempting to reclassify cerebrovascular lesions. Current methods for neuropathological characterization of these lesions differ little to those used a century ago, in contrast to the advances seen in methods employed for detecting other brain-related conditions such neoplasias and neurodegenerative diseases. Therefore, methodological improvement is an essential first step. This can be achieved through the development of antibodies against certain markers expressed during cerebrovascular lesions or by introducing imaging techniques into pathology labs.

A pathogenic-based classification will be beneficial in establishing preventive and also therapeutic measures against these lesions, and should be prioritized in future investigations on vascular dementia.
